# Choosing Anthropometric Indicators to Monitor the Response to Treatment for Severe Acute Malnutrition in Rural Southern Ethiopia—Empirical Evidence

**DOI:** 10.3390/nu9121339

**Published:** 2017-12-08

**Authors:** Amare Worku Tadesse, Elazar Tadesse, Yemane Berhane, Eva-Charlotte Ekström

**Affiliations:** 1 Department of Women’s and Children’s Health, International Maternal and Child Health Uppsala University, SE-75185 Uppsala, Sweden; elazar.balla@kbh.uu.se (E.T.); Lotta.Ekstrom@kbh.uu.se (E.-C.E.); 2 Addis Continental Institute of Public Health, P.O. Box 26751/1000 Addis Ababa, Ethiopia; yemaneberhane@gmail.com

**Keywords:** anthropometric indicators, monitor response to treatment, severe acute malnutrition, children

## Abstract

The World Health Organization (WHO) recommends the assessment of nutritional recovery using the same anthropometric indicator that was used to diagnose severe acute malnutrition (SAM) in children. However, related empirical evidence from low-income countries is lacking. Non-oedematous children (*n* = 661) aged 6–59 months admitted to a community-based outpatient therapeutic program for SAM in rural southern Ethiopia were studied. The response to treatment in children admitted to the program based on the mid-upper arm circumference (MUAC) measurement was defined by calculating the gains in average MUAC and weight during the first four weeks of treatment. The children showed significant anthropometric changes only when assessed with the same anthropometric indicator used to define SAM at admission. Children with the lowest MUAC at admission showed a significant gain in MUAC but not weight, and children with the lowest weight-for-height/length (WHZ) showed a significant gain in weight but not MUAC. The response to treatment was largest for children with the lowest anthropometric status at admission in either measurement. MUAC and weight gain are two independent anthropometric measures that can be used to monitor sufficient recovery in children treated for SAM. This study provides empirical evidence from a low-income country to support the recent World Health Organization recommendation.

## 1. Introduction

Severe acute malnutrition (SAM) is estimated to cause 540,000 child deaths every year [1]. Since being introduced in 2007, the community management of SAM (CMAM) is the recommended treatment guideline, which combines an outpatient therapeutic program (OTP) with ready-to-use therapeutic foods (RUTF) for uncomplicated cases, and inpatient treatment for complicated cases [2,3]. The introduction of the OTP has improved the coverage, access, and cost-effectiveness of SAM management [3,4,5]. 

The current guidelines recommend the use of values of mid-upper arm circumference (MUAC) lower than 115 mm or of weight-for-height/length (WHZ) below the −3 *Z* score of the WHO standard [2,3,6] as two independent anthropometric criteria for identifying children with SAM for treatment [7]. Although both indicators are commonly used in nutritional rehabilitative programs, WHZ and MUAC were shown to identify different sets of children as having SAM [2,8,9,10]. Such discrepancy was also shown to vary in different settings [7,8] and according to the age and sex of the children identified as having SAM [10]. Although both indicators measure the nutritional status [6,11], uncertainty exists about the appropriate indicator to use for monitoring the response to treatment, recovery, and discharge from programs providing care for SAM.

When the admission was based on WHZ, the indicators to define recovery from SAM used to be based on WHZ [12]. For programs using MUAC as the admission criteria, the WHZ-based discharge criteria were not applicable as some children might have already fulfilled the WHZ discharge criteria at the time of admission into the program [2,13]. To avoid this problem and to eliminate the need for height measurement, the discharge criterion was redefined to be based on the percentage weight gain since admission [14]. However, the use of the percent weight gain was problematic because it led the most severely malnourished, and hence lighter, children to require a smaller absolute weight gain to meet the discharge criteria despite the risk of insufficient recovery [15,16,17]. Currently, the WHO guidelines on SAM management recommends that the criteria for discharging children from the treatment should be based on the same indicator that was used to diagnose SAM [7]. 

The response to treatment of children with SAM admitted to programs on the basis of WHZ is well documented [5,18,19,20,21], and weight gain has been the indicator of choice for monitoring the response to treatment for SAM [2,11]. The application of the standard protocol that uses the same measure for both the admission and discharge criteria aims to enable sufficient nutritional rehabilitation and to reduce mortality in children with SAM. The follow-up of severely malnourished children on the basis of MUAC alone was used in some settings [16,17], and this strategy is now included in the WHO updated recommendations [7]. However, there is limited evidence that the mid-upper arm circumference is an indicator of the progress in recovery during nutritional rehabilitation.

A good monitoring indicator needs to be responsive to the changes in the nutritional status and should predict a negative or positive outcome. The decision to use either the weight gain or the MUAC change as an indicator for monitoring the response to treatment in programs providing care for SAM needs to be based on understanding the relationship between these indicators to ensure that sufficient nutritional rehabilitation is achieved during the treatment of SAM children in the OTP. It is also imperative to generate empirical evidence in support of the current treatment guidelines [7]. Thus, we analyzed the average MUAC and weight gains of SAM children admitted to the OTP after 4 weeks of rehabilitation.

## 2. Methods 

### 2.1. Study Design and Setting

This cohort study was part of a larger study (COMSAM) which aimed at evaluating the effectiveness of a community-based management of a severe acute malnutrition program in four adjacent districts in southern Ethiopia. The details of the study were described previously [15,22], but a brief description is provided here. In Ethiopia, the OTP has been scaled up and integrated into the existing public health system, including health posts (the lowest tier in the Ethiopian health system hierarchy). As part of CMAM, the children in the study area are screened for signs of malnutrition by community health workers. The OTP offers services to severely malnourished children aged 6–59 months. According to the Ethiopian national SAM management protocol [23], the children are admitted to the programme when their MUAC is lower than 11 cm or nutritional oedema is present. After admission, the children receive RUTF based on their weight on a weekly basis and additional supportive therapies based on their needs [23]. The study area is known for its recurrent nutritional emergencies and chronic food insecurity [24]. As a result, severe acute malnutrition in children is highly prevalent in the districts, and child mortality is also reported to be one of the highest in this part of the country [25].

### 2.2. Participants and Data Collection 

The children admitted to the OTP in health posts of the study districts were identified using the health post registry. The research team collected independent anthropometric measurements, including mid-upper arm circumference, weight, height, and presence of bilateral oedema within one week of admission and 4 weeks after admission. Follow-up measurements were taken at the children’s homes. The mid-upper arm circumference of children aged 6 to 59 months was measured. Oedema on the feet was checked in children by pressing the foot. Other household, caregiver, and child information was also collected through household interviews conducted during these home visits [15,22]. 

**Figure 1:**
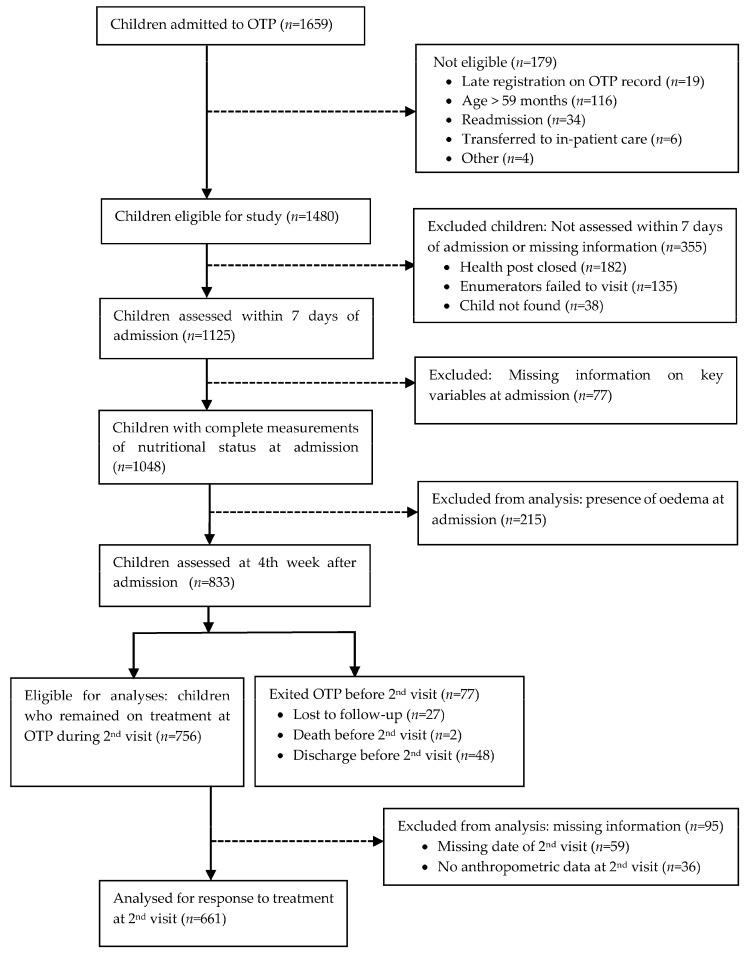
Flowchart of children admitted to the outpatient therapeutic program (OTP).

In this paper, we used a subset of children admitted to the OTP during a 6 months period, from July to December 2011 (*n* = 1048), who had complete information on anthropometry at admission. Children with oedema at admission or follow-up were excluded from the analyses, as the changes in the anthropometric measurements could be obscure because of the fluid retained in the body [13]. Further, only children whose anthropometric measurements were taken at the 4th week of follow-up (*n* = 661) were included in the analysis for the assessment of the response to treatment at the 4th week of follow-up (Figure 1).

### 2.3. Measures

#### 2.3.1. Anthropometry

The weight of each child was measured to the nearest 0.1 kg using the UNICEF electronic scale. Their MUAC was taken using the WHO-recommended MUAC tape and procedure, and their length and height were measured to the nearest 0.1 cm using the UNICEF recommended model of the wooden board, as per the WHO protocol [26,27]. Nutritional indices of malnutrition were calculated using the WHO Anthro (Version 3.2.2, WHO, Geneva, Switzerland) to produce the *Z*-scores for weight and length and height data, using the 2006 WHO Growth Standards [28]. To set categories based on the nutritional status at admission and distinguish between the gains in MUAC or weight that occurred during the process of normal growth and response to treatment, MUAC measurements and WHZ-scores were categorized into three groups, i.e, MUAC < 110 mm, 110–114 mm, and ≥115 mm; and WHZ < −3, −3 ≤ WHZ < −2 and WHZ ≥ −2, respectively. 

#### 2.3.2. Outcome

The response to treatment was defined by two anthropometric indicators: the average MUAC gain per day and the average weight gain per day. The average MUAC gain, expressed as mm/day, was calculated by taking the difference in the MUAC measurements between the admission and the 2nd visit conducted after 4 weeks of follow-up in the programme and dividing this value by the total number of days between each measurement. The average weight gain, expressed as g/kg/day, was calculated by dividing the rate of weight gain between the admission and the 4th week of follow-up by the child’s average weight, in line with the recommendations [27].

#### 2.3.3. Sociodemographic Characteristics 

Data on housing quality, caregiver characteristics such as occupation, education, and age (years), child age, child sex, and child length and height were collected. The housing quality was used as a proxy indicator of the household’s overall material resources. This measure was based on the type of material used in the construction of the house, the sources of drinking water, and the latrine facilities. Each characteristic was coded from 1 to 3, where a value of “1” referred to the lowest housing quality and a value of “3” to the highest. The household variables were summed up to create a housing quality score ranging from 3 to 9, which was further divided into tertiles to define the lowest, middle, and highest housing quality level.

### 2.4. Statistical Analysis

Descriptive statistics, including proportions and 95% confidence intervals, were computed to describe age and sex distributions according to the children’s nutritional status at admission and the change in anthropometric indicators. The ages of the children were dichotomized to correspond to categories usually used in field nutrition programs: young (age < 24 months) and older children (age ≥ 24 months). We created continuous variables to define the response to treatment by using average MUAC and average weight gain values, as described above. We used the general linear model (GLM) to estimate the gain in anthropometric indicators (as continuous outcome variables) according to MUAC and WHZ at admission.

Based on the UNICEF conceptual model of child growth and development [29] as well as on previous research on the determinants of treatment outcome for the OTP [30,31,32], certain child, caregiver, and household characteristics that can influence child growth and nutrition were selected as potential confounders. Three statistical models were then created: the first unadjusted, the second adjusted for child age, sex, and length and height, and the third adjusted for housing quality, caregiver characteristics (occupation, age in years, education), and duration of the follow-up until the 2nd visit, in addition to the child characteristics. The regression diagnostic procedure and visual impressions of the standard errors of the regression coefficients in the multivariable model showed no evidence of multicollinearity (variance inflation factor < 1 and tolerance > 0.9). The data were analysed using SPSS 20 (International Business Machines Corporation, New York, NY, USA) statistical software package for Windows.

### 2.5. Ethics

Ethical approval was received from the institutional ethical review board of the Addis Continental Institute of Public Health (ACIPH), Ethiopia, and the regional ethical review board in Uppsala, Sweden. Permission to conduct the study was obtained from regional and district health offices. The Declaration of Helsinki was followed when conducting the study. A verbal informed consent was obtained from the caregiver of the child prior to conducting the interviews.

## 3. Results

Figure 1 illustrates the complete participant flow from admission to 4 weeks of therapy in the OTP. Of 1659 children admitted to the OTP, 179 were excluded because ineligible and 355 because their nutritional status was not assessed within seven days of admission. After assessing the nutritional status of 1125 children, 77 children with missing key information at admission and 215 children with oedema were excluded from the analysis in this study. Of 833 children who were followed for 4 weeks, 77 exited from the OTP before the 4th week follow-up assessment by the study team. Of the 756 eligible children for this study, 661 children with a complete follow-up at 4 weeks were included in the analysis. We compared the excluded group of children with that of children included in this study and found no significant differences in their ages or sex (for those with available information) or in the caregivers’ and household characteristics of the children. The background household, caregiver, and child characteristics of those who participated in the OTP are shown in Table 1. Nearly three out of four households had a house with a thatched roof with wood and mud/grass walls, open-pit latrine, and access to a protected source of drinking water. A majority of the mothers were married, aged between 20 and 39 years, and had at least some form of primary education. About 60% of the children were females and 70% were under two years of age.

The nutritional status at admission was analysed for 661 children who were admitted to the OTP based on mid-upper arm circumference (MUAC) criteria and who received 4 weeks of therapy. When using the MUAC criteria to define SAM, 75.9% had SAM on admission (57% MUAC < 110 mm and 18.9% MUAC 110–114 mm) and 24% did not have SAM. When using the WHZ criteria to define SAM, 28.1% had SAM and 35.6% did not have SAM. MUAC classified a higher proportion of young children (age < 24 months) compared to the older children (age ≥ 24 months) as severely malnourished. Among the studied children, WHZ classified a higher proportion of boys compared to girls as severely malnourished. Thus, the two indicators classify children in a significantly different way (Table 2).

MUAC and weight increased in all children overtime. However, the average gain for these measures varied between children with different degrees of severity of malnutrition as well as whether the indicator used was based on MUAC or WHZ. The average MUAC and weight gains after 4 weeks of follow-up were 0.17 mm/day (±0.2 SD) and 1.8 g/kg/day (±4.0 SD), respectively (Table 3). When MUAC was used to define SAM at admission, MUAC gain was significantly greater among severely malnourished children (MUAC < 110 mm) compared with children with no SAM (MUAC ≥ 115 mm). Similarly, when WHZ was used to define SAM at admission, weight gain was significantly greater among severely malnourished children (WHZ < −3) compared with children with no SAM (WHZ ≥ −2). The anthropometric changes were marginal for those malnourished children with MUAC 110–114 mm and −3 ≤ WHZ < −2, compared to children with no SAM (MUAC ≥ 115 mm and WHZ ≥ −2). The average gain was two-fold for MUAC (0.20; 95% CI 0.17, 0.23) and three-fold for weight (3.1; 95% CI 2.4, 3.8) (Table 3).

Children with the lowest MUAC (MUAC < 110 mm) showed a significant change on the average MUAC gain after 4 weeks of therapy but no significant difference in weight gain. Children with the lowest WHZ (WHZ < −3) showed a significant change on the average weight gain after 4 weeks of therapy but no significant difference in the average MUAC gain (Table 4). Thus, both MUAC and weight gain detected a good response to treatment in 4 weeks, and the largest response was observed among the severely malnourished children. However, using a measure different from the one used to define SAM at admission did not allow to detect a significant change in the response to treatment.

## 4. Discussion

In this cohort study, we found that SAM children showed changes in both MUAC and weight measurements following treatment. For the MUAC-based definition of SAM at admission, the gain in MUAC was the indicator of choice to monitor the treatment response in children treated for SAM. Likewise, when WHZ was used to define SAM at admission, weight gain was the indicator used to monitor the response to treatment.

An average MUAC gain of 0.17 mm/day and weight gain of 1.8 g/kg/day for the children in our cohort compared unfavorably to those reported by other community-based nutritional programs, where the average MUAC and weight gains for children recovering from acute malnutrition varied between 0.2 to 0.52 mm/day [17,33] and 3 to 6.8 g/kg/day [33,34], respectively. The lower MUAC and weight gain compared to the recommended standards and other studies [17,35] may possibly be explained by differences in the adherence to optimal management of children with SAM under the OTP. Low frequency of feeding per day and sharing of the RUTF with other members of the household are likely to cause poor recovery of the children treated at home [22,34,36]. Results from our previous study area also indicated that the sharing of RUTF with other family members, mostly children, was justified by social norms favouring food sharing, shortage of food in the household, the good taste of RUTF, and its high-energy properties [22,36]. The lower MUAC and weight gains may also be due to the providers’ nonadherence to the guidelines on SAM management where the follow-up of the children being managed at the OTP was inadequate in relation to the monitoring of the children’s response to treatment and the provision of the recommended amount of RUTF [7,22,36].

In our study, the rate of MUAC gain was the largest in children with the smallest MUAC at admission. This gain could reveal, besides the normal physiologic growth, changes in the lean tissue during recovery [37,38]. Thus, children with low MUAC had a larger gain in MUAC, suggesting that they experienced rapid catch-up growth in response to a therapeutic diet [16,17]. Our results also show that children with the lowest WHZ at admission had higher proportional weight gain than the children with a higher WHZ at admission. A rapid weight gain happens during recovery for children with a deficit in WHZ [39], whereas the rates of weight gain fall substantially when children approach a normal WHZ [39]. However, our findings on the gains in MUAC and weight differed according to the indicator that was used to define SAM at admission. These gains are likely to reflect normal growth during rehabilitation to achieve sufficient recovery from SAM [13]. Thus, it is worth noting the importance of using the same indicator to monitor the response to treatment as the one used on admission for diagnosing SAM. Moreover, our analysis showed no effect of child age, sex, and length and height on the rate of weight or MUAC gain (Table A1), which may support a reasonable argument that MUAC and weight may be used independently to monitor the response to treatment.

In children being treated for SAM in OTPs, the gains in MUAC and weight were shown to be closely correlated and follow a similar recovery trajectory [40]. As weight loss or the failure to gain weight in children may suggest that follow-up in the community or referral to hospital are necessary [7,40], the failure to gain MUAC in children may similarly be indicative of the need for such a procedure. Our findings are also in line with the results of the above studies. Furthermore, using the same measurement tool for admission, treatment monitoring, follow-up, and discharge could make programs more coherent and understandable to caregivers [17].

Decreasing the growth velocity measures based on weight and MUAC were shown to be useful in identifying children at high risk of dying [41,42,43,44,45]. The application of these measures in the field by minimally trained community health workers is a challenge, as calculating the growth velocity requires mathematical skills similar to those used for completing growth charts [13]. Decreased gains in weight and MUAC could reflect a recent drop in the growth curve and could possibly identify children at an increased risk of death [44]. However, it may be possible to develop simpler monitoring tools and protocols for the appropriate management of SAM based on changes in MUAC and weight with field testing and refinement [41], as the children’s nutritional status is typically monitored for progress at the OTP [13,17]. 

In anthropometric assessment, the reliability of the measurements is an important requirement as it directly influences the admission and discharge criteria for children in nutrition interventions [46]. The reliability reflects the repeatability of the results when several measurements are performed by the same (intraobserver) or different (interobserver) observers under the same conditions [47]. Previous studies of reliability were conducted within a carefully controlled hospital or research environment and reported high reliability scores for absolute measures of weight, length and height, and MUAC [48,49]. As variations in the reliability of the measurements could influence the interpretation of the children’s responses to the treatment of SAM, all enumerators went through training to ensure the standardization of the measurements, and used the WHO-recommended MUAC tapes and the UNICEF-recommended weight and height boards. A rigorous measurement technique is a means to minimize random errors in anthropometric measurements and the regression to the mean and thus to reduce the risk of insufficient reliability as an explanation to our findings. 

A potential limitation of this study was that the analysis was based on data collected from children admitted to the OTP by MUAC criteria. However, because there was no significant difference in terms of their background characteristics in the proportion of children with low WHZ between the population [10] and the study sample, our estimation of the gains in anthropometric measures is likely to reflect a valid estimate of the response in the children treated for SAM.

## 5. Conclusions

The gains in MUAC and weight are two independent anthropometric measures that can be used to monitor the response to treatment for severe acute malnutrition and ensure sufficient recovery. This study provides empirical evidence that supports the recent recommendation to use the same anthropometric indicator to identify children for the treatment of SAM and to monitor their response to nutritional rehabilitation programs.

## Figures and Tables

**Table 1:** Household, caregiver, and child characteristics of children admitted to the OTP.

**Household Characteristics**	***n*** **(%)**
Sanitation (*n* = 660)	
Pit latrine with slab	17 (2.6)
Open pit	510 (77.2)
Open space (Bush/farm land, other)	123 (20.2)
Source of drinking water (*n* = 659)	
Protected source ( public tap, protected well/spring)	501 (76.0)
Unprotected source (spring, well, other)	158 (24.0)
House construction (*n* = 660)	
Corrugated iron roof with wood and mud wall	168 (25.5)
Thatched roof with wood and mud/grass wall	492 (74.6)
Number of children under five in the household (*n* = 661)	
One	396 (59.9)
More than one	265 (40.1)
**Caregiver Characterstics**	
Relationship to child (*n* = 660)	
Biological mother	586 (88.8)
Marital status (*n* = 638)	
Married	575 (90.1)
Age (in years) (*n* = 653)	
15–19	9 (1.4)
20–29	268 (41.0)
30–39	314 (48.1)
≥40	62 (9.5)
Current occupation (*n* = 661)	
Nonsalaried job	96 (14.5)
Farmer	349 (52.8)
Petty trade & wage work	216 (32.7)
Educational status (*n* = 661)	
Never attended school	20 (3.0)
In but did not complete primary school	355 (69.4)
Completed primary and above	182 (27.6)
**Child Characteristics**	
Sex (*n* = 661)	
Female	389 (58.9)
Age (in Months) (*n* = 661)	
6–11 months	289 (43.7)
12–23 months	207 (31.3)
24–35 months	66 (10.0)
36–47 months	63 (9.5)
48–59 months	36 (5.4)

**Table 2:** MUAC and WHZ of children categorized by sex and age at admission to the outpatient therapeutic program.

		MUAC at Admission									WHZ at Admission								
		MUAC < 110 mm			MUAC 110–114 mm			MUAC ≥ 115 mm			WHZ < −3			−3 ≤ WHZ < −2			WHZ ≥ −2		
	*N*	*n*	%	95% CI	*n*	%	95% CI	*n*	%	95% CI	*n*	%	95% CI	*n*	%	95% CI	*n*	%	95% CI
Sex																			
Girls	389	228	58.6	53.5, 63.5	71	18.3	14.6, 22.5	90	23.1	19.1, 27.7	88	22.6	18.6, 27.2	148	38.1	33.2, 43.1	153	39.3	34.5, 44.4
Boys	272	149	54.8	48.7, 60.8	54	19.9	15.4, 25.2	69	25.3	20.4, 31.1	98	36.1	30.4, 42.1	92	33.8	28.3, 39.8	82	30.1	24.8, 36.0
Age																			
<24 months	496	310	62.5	58.1, 66.8	86	17.3	14.2, 21.0	100	20.2	16.8, 24.0	146	29.4	25.5, 33.7	186	37.5	33.3, 41.9	164	33.1	29.0, 37.4
≥24 months	165	67	40.6	33.1, 48.5	39	23.6	17.5, 31.0	59	35.8	28.6, 43.6	40	24.2	18.1, 31.6	54	32.7	25.8, 40.5	71	43.1	35.4, 51.0

**Table 3:** Average MUAC gain and weight gain of children categorized by their anthropometric status at admission to the outpatient therapeutic program after 4 weeks of therapy.

	Average MUAC Gain, mm/day		Average Weight Gain, g/kg/day	
	Mean	95% CI	Mean	95% CI
All children	0.17	0.15, 0.19	1.8	1.5, 2.2
**MUAC at admission**				
MUAC < 110 mm	0.20	0.17, 0.23	2.0	1.5, 2.4
MUAC 110–114 mm	0.17	0.13, 0.21	1.9	1.4, 2.4
MUAC ≥ 115 mm	0.10	0.04, 0.13	1.5	0.8, 2.1
**WHZ at admission**				
WHZ < −3	0.17	0.12, 0.22	3.1	2.4, 3.8
−3 ≤ WHZ < −2	0.17	0.13, 0.20	1.7	1.3, 2.2
WHZ ≥ −2	0.17	0.14, 0.20	0.9	0.5, 1.4

**Table 4:** GLM results for the estimated effect of the anthropometric status at admission on average MUAC and weight gains after 4 weeks of follow-up.

	**Average MUAC Gain (mm/day)**								
**Anthropometric Status at Admission**	**Unadjusted**			**Model I ^†^**			**Model II ^‡^**		
	**β**	**95% CI**	***R*^2^**	**β**	**95% CI**	***R*^2^**	**β**	**95% CI**	***R*^2^**
MUAC < 110 mm	0.12 **	0.07, 0.17	0.031	0.14 **	0.09, 0.20	0.046	0.14 **	0.09, 0.20	0.063
MUAC 110–114 mm	0.09 **	0.02, 0.15		0.09 **	0.03, 0.16		0.10 **	0.04, 0.17	
MUAC ≥ 115 mm	Ref			Ref			Ref		
	**Average Weight Gain (g/kg/day)**								
**Anthropometric Status at Admission**	**Unadjusted**			**Model I ^†^**			**Model II ^‡^**		
	**β**	**95% CI**	***R*^2^**	**β**	**95% CI**	***R*^2^**	**β**	**95% CI**	***R*^2^**
WHZ < −3	2.16 **	1.37, 2.95	0.042	2.16 **	1.34, 2.96	0.043	2.17 **	1.34, 3.00	0.054
−3 ≤ WHZ < −2	0.81 *	0.07, 1.55		0.79 *	0.05, 1.54		0.81 *	0.05, 1.57	
WHZ ≥ −2		Ref			Ref			Ref	

The regression coefficients (β) represent the mean increase in the dependent variables (average MUAC and weight gain) between the categories of the independent variables. MUAC, mid upper circumference; WHZ, weight-for-height *Z*-score; Ref, reference category; * *p* < 0.05, ** *p* < 0.01; ^†^ Adjusted for child sex, age (months), and length and height; ^‡^ Adjusted for housing quality, caregiver characteristics (occupation, education, and age in years), duration of follow-up, child sex, child age (months) and child length and height using the general linear model (GLM).

## Appendix A

**Table A1:** GLM results for the estimated effect of child, caregiver, and household characteristics on average MUAC and weight gain after 4 weeks of follow-up.

	Average MUAC Gain (mm/day)		Average Weight Gain (g/kg/day)	
Variables	β	95% CI	β	95% CI
Housing quality				
Lowest	0.13	−0.11, 0.38	0.87	−2.80, 4.54
Middle	0.14	−0.11, 0.39	0.89	−2.84, 4.62
Highest	Ref		Ref	
Caregivers’ education				
Never attended school	−0.07	−0.20, 0.06	−1.40	−3.35, 0.55
In but did not complete primary school	−0.01	−0.06, 0.04	−0.01	−0.76, 0.74
Completed primary school or above	Ref		Ref	
Caregivers’ occupation				
Non-salaried	0.08	−0.02, 0.15	−0.01	−1.03, 0.99
Farmer	0.03	−0.14, 0.81	0.27	−0.45, 0.99
Petty trade & wage work	Ref		Ref	
Caregivers’ age (years)	0.001	−0.01, 0.01	−0.04	−0.09, 0.01
Duration of follow up	−0.005	−0.01, 0.01	−0.06	−0.15, 0.03
Child age				
<24 months	−0.03	−0.09, 0.03	−0.12	−1.12, 0.88
≥24 months	Ref		Ref	
Child sex				
Boys	−0.002	−0.05, 0.04	−0.14	−0.80, 0.53
Girls	Ref		Ref	
Child length/height	0.003	−0.01, 0.01	−0.02	−0.07, 0.03

β, Coefficients for variables in final model (GLM).
